# Laudator Temporis Acti

**Published:** 1905-08-26

**Authors:** 


### Hospital Clinics.

### LAUDATOR TEMPORIS ACTI.

Dr. F. Graham Crookshank is something of a pessimist and something of a cynic, a dangerous combination for a medical man, as is evident from parts of a very readable paper contributed to the *Public Health* issue for August. He bewails the loss of the old "barn-like structure in which Liston, Erichsen, and Thompson had operated and Jenner, Quain, and Reynolds taught," to wit, of course, University College Hospital. "But this barn-like structure has disappeared, and, surrounded by a wilderness of chimney-pots, there stands in its place a palace of red brick and terra-cotta, with turrets, pinnacles, and flying bridges, with marble halls and crystal corridors, to use the cant of specification, 'all embellished with enrichments of approved design.' Here one sees gynæcologists dip their elbows in silver bowls that might have adorned Solomon's temple; here students imbibe instruction perched on marble slabs like oysters at the fishmonger's; here surgeons turn taps on with their feet, and here, on metal trolleys, patients whizz to and fro the theatre like chops on a hot-water dish. It is all very wonderful, but is it really right to spend half a million of money that in Smithfield and Gower Street successes be obtained which, by the observation of a few simple rules, Alanson commanded at Liverpool one hundred and twenty-five years ago in a measure that in our time has never been surpassed and seldom equalled? It is no doubt an admirable thing to see a gynæcologist lave his elbows in an inverted dish-cover that cost fifty guineas. But might it not be better sometimes, for fifty pence, to take the patient to where there are fewer microbes, and perhaps fewer gynæcologists?"

Dr. Crookshank is a medical officer of health, and a gentleman of wide experience in his profession, and we incline to expect some weight in his pronouncements. We read through again the above journalistic excursion, and became persuaded of two things—(1) The author does not like gynæcologists; (2) He regrets the rebuilding of University College Hospital. We are not admitted to the explanation of his dislike for gynæcologists, but his regret at the rebuilding appears to depend upon the fact that someone from that palatial hospital has told him "that he is out of date; that sewer-gas is a beneficent afflatus; that typhoid is always due to oysters or watercress, and has nothing to do with drains; and that so far from tuberculosis having anything to do with wet soil, overcrowding, dirt, and foul air, it is really a matter best dealt with by little placards in the tramcars, that he who rides and chews may read and not spit."

The unjudicial attitude of the writer is well-evidenced by this rhetorical outburst; it cannot be believed, in the absence of a more definite statement, that anyone has really told him all these things, but it is an approved method of advocacy to put opinions in the mouth of an adversary for the purpose of giving them the lie. None the less, it is disappointing to find these words from the pen of an experienced member of the profession, when for years one has hardly been able to find a medical publication in which stress is not laid upon the overwhelming importance of ventilation in the prevention of tuberculosis. On the strength of a dictum of Sir Edward Fry, the author falls foul of "one of our most eminent pathologists" for stating baldly that "tuberculosis is a disease caused by the bacillus tuberculosis." Surely a pitiful appeal to casuistry, since one might have assumed the postulate that no infection can take place except in the case of a susceptible subject. It is arrogant and superior, in the author's view, to speak of "chronic pulmonary tuberculosis" in place of "pulmonary phthisis," and this in spite of the fact that there are forms of pulmonary phthisis which are not tuberculous. The doctrine that tuberculosis is propagated by spitting, rests, we are told, on the thesis that the bacillus tuberculosis is an obligatory parasite: a masterpiece of faulty logic, since, though they be facultative saprophytes to never so high a degree, the more bacilli of tuberculosis put into circulation, the greater the chance of infection of susceptible subjects, and this apart from the known enhancement of virulence produced by "passage" through susceptible soils.

In attempting the *rôle* of an open-minded observer Dr. Crookshank has avowed himself a foe of accuracy, even in so far as it is attainable. Clinical evidence satisfies him: if a case presents the features of typhoid fever, it is typhoid fever, whatever the bacteriologists say, as though the κρίσις χαλεπὴ were not as old as Hippocrates. Here is a paragraph which will appeal to clinical bacteriologists. "I know that the worst attack of ocular, palatal, respiratory and crural paralysis that I have known occurred in a lady who, never associated with children or cats, when a pond near to her house was drained, suffered from a membranous tonsillitis *in which the Klebs-Löffler bacillus had no part, and the bacillus subtilis was present in overwhelming force.*" The italics are ours and indicate the fallacy. To search for diphtheria bacilli amid a rank growth of bacillus subtilis is an idle task, and he is a bold and imprudent scientist who would venture an opinion from such a culture.

Because those who pursue accurate bacteriological methods and have learnt to believe in them, claim to know something, it does not follow that they claim to know everything, as Dr. Crookshank would have us believe. The bacteriological school would be the first to confess their ignorance of the origin of pathogenicity in micro-organisms, and the laws governing its variation. But it is a strange proposition that we should neglect to apply the knowledge we have because it is limited. Dr. Crookshank has missed several opportunities of convincing sceptics. He details a diphtheria epidemic which, after resisting all the approved prophylactic measures of bacteriologists, yielded to the drainage of a previously unsuspected well, situated beneath one of the class-rooms. The result of cultivation of that well water would have been of great interest. Again, he instances as a case of typhoid infection *de novo*, a man living in an isolated spot near a cess-pool. We are told that the appearances were clinically typical, but he does not say which of the variably associated symptoms of typhoid fever justify that statement. Widal's reaction was not employed, apparently, nor are we told whether an autopsy confirmed the diagnosis, and thus the value of the case is hopelessly stultified to any but the extreme members of the "common-sense, old-fashioned" school to which Dr. Crookshank belongs.

